# Histological staining of tick bite skin biopsies for spirochetes and Powassan virus RNA

**DOI:** 10.1128/spectrum.00902-24

**Published:** 2025-01-10

**Authors:** Jihane Oufattole, Anne Piantadosi, Sam R. Telford, Alvaro C. Laga, Isaac H. Solomon

**Affiliations:** 1 Department of Pathology, Brigham and Women’s Hospital, Boston, Massachusetts, USA; 2 Harvard Medical School, Boston, Massachusetts, USA; 3 Department of Pathology and Laboratory Medicine, Emory University School of Medicine, Atlanta, Georgia, USA; 4 Department of Infectious Disease and Global Health, Tufts Lyme Disease Initiative, Tufts University, North Grafton, Massachusetts, USA; David Geffen School of Medicine at UCLA, Los Angeles, California, USA

**Keywords:** tick-borne infection, Lyme disease, Powassan virus, immunohistochemistry, *in situ *hybridization

## Abstract

*Ixodes scapularis* is a vector of several human pathogens in the United States, including *Borrelia burgdorferi,* the cause of Lyme disease, and Powassan virus (POWV), an emerging cause of severe encephalitis. Skin biopsies from tick bite sites are frequently collected and tested for the presence of spirochetes (*Borrelia* spp.), which remain elusive. POWV testing is not performed despite the increasing incidence of POWV encephalitis. To determine the utility of staining skin biopsies for tick-borne pathogens, formalin-fixed paraffin-embedded specimens from 2010 to 2023 were examined by hematoxylin and eosin, Warthin–Starry stain, spirochete immunohistochemistry, and POWV *in situ* hybridization (ISH). These assays were optimized by staining histological sections from whole ticks, from which spirochetes were detected in two out of 14 patient-derived specimens, and POWV RNA was detected in multiple experimentally infected ticks. None of the 36 skin biopsies were positive for spirochetes, including 20 with retained tick mouth parts and six from patients with positive Lyme serology. POWV ISH was negative in all biopsies, including one laboratory confirmed case. Combining patients with skin biopsies and whole ticks (*n* = 50), spirochetes were detected in specimens from only 1/5 (20%) patients with suspected erythema chronicum migrans (ECM). Although limited, our data suggest a poor correlation between ECM and the detection of spirochetes, findings in concordance with the published literature. Similarly, the absence of detectable POWV RNA in any of the tested skin biopsies or patient-derived ticks, including one laboratory confirmed case, suggest the limited utility of POWV ISH for clinical use without further analysis with a larger sample size.

**IMPORTANCE:**

Tick-borne infections, including Lyme and Powassan encephalitis, cause significant morbidity and mortality and are challenging to diagnose and study in humans. We examined skin biopsies from patients with tick bites to look for direct evidence of microbes by histochemical, immunohistochemical, and *in situ* hybridization stains. To validate these assays, we also tested the same stains on histological sections from whole ticks infected with spirochetes or Powassan virus. Examination of skin biopsies using similar tools may prove valuable in studying the pathogenesis of diseases, such as southern tick-associated rash illness, for which a causative pathogen has not yet been identified.

## INTRODUCTION

Ticks, which are small hematophagous arthropods, play a crucial role as vectors for a diverse array of bacterial, viral, and parasitic pathogens capable of causing severe illness and death in humans. Detecting these pathogens accurately and promptly is essential for effective disease prevention and treatment. Although various diagnostic tests are used to screen for tick-borne diseases, each method has its limitations (1).

Lyme disease caused by *Borrelia burgdorferi* and transmitted by *Ixodes scapularis* (blacklegged tick or deer tick) is the most prevalent vector-borne disease in the USA, with an estimated 465,000 cases annually, concentrated primarily in the Northeast, Mid-Atlantic, and Midwest regions (2). Diagnosis requires a nuanced approach based on the disease stage and regional prevalence. In endemic areas, a patient with early localized disease characterized by the distinctive erythema chronicum migrans (ECM) should be diagnosed clinically, as serologic testing tends to be insensitive during this stage (3, 4). Serologic testing is also not recommended for patients with a low pre-test probability, including asymptomatic individuals and those with non-specific symptoms, due to the risk of misinterpretation leading to unnecessary treatment (5). The Centers for Disease Control and Prevention (CDC) endorse a two-tiered serologic testing strategy, including an initial enzyme immunoassay or immunofluorescence assay, followed by a western blot, although it can be difficult to distinguish between past and current infections in advanced stages of Lyme disease (6–8). PCR testing on blood or skin samples also poses limitations, with false positives due to nucleic acid contamination and false negatives due to variations in target gene sequences (9, 10). While Lyme disease can be clinically diagnosed and treated based on ECM in the appropriate clinical context, skin biopsies are commonly obtained from known and suspected tick bite sites in the absence of ECM. Biopsies may reveal distinctive features of tick bite, such as tick mouthparts, vascular hyaline thrombi, and the characteristic wedge-shaped perivascular interstitial infiltrate with eosinophils observed in reactions to arthropod bites (11).

Powassan virus (POWV) is an emerging tick-borne flavivirus most prevalent in the Northeastern and Great Lakes regions of the USA (12). POWV lineage II, also referred to as deer tick virus (DTV), raises particular concern, as it is transmitted by *I. scapularis*, the primary vector for *B. burgdorferi* (13). DTV is found in 1–4% of ticks in the Northeast USA (13–15). POWV has been increasingly identified as a cause of encephalitis, with 218 cases of human disease reported to the CDC between 2015 and 2022, a major escalation from the 69 cases documented in the preceding 8-year period from 2007 to 2014 (16). In 10% of cases, POWV infection in humans leads to a febrile illness with progressive neurological symptoms, often resulting in death, and more than 50% of survivors endure severe and permanent neurological complications (17–19). Whether POWV causes non-neuroinvasive disease is currently under investigation. Diagnosing POWV infection requires detection of IgM and neutralizing virus-specific antibodies in the serum or cerebrospinal fluid (CSF), detection of a four-fold increase in serum virus-specific antibodies, or isolation of viral antigen or nucleic acid from bodily tissues (20). Laboratory diagnosis is complicated by the transient nature of the viremic phase, the delayed detectability of antibodies in early disease stages, and the lack of widespread availability of POWV-specific assays (21). POWV antibody detection from serum and CSF, which was only available from public health laboratories until recently, is typically the preferred method of diagnosis after 1 week of infection but is frequently negative in B-cell depleted patients who present with the most severe disease (22). POWV RT-PCR testing from blood and CSF has also been limited to public health laboratories until recently and is generally negative after the first week of infection before most patients present for medical care, with the exception of immunosuppressed patients with prolonged viremia. RT-PCR can be falsely negative if probes are not optimized for currently circulating strains. Unbiased metagenomic next-generation sequencing from the CSF available from multiple academic/commercial laboratories can effectively detect POWV but is significantly more costly than other targeted methods (23). In mouse models of infection, *in situ* hybridization (ISH) has proven useful in identifying POWV RNA at the tick-bite site; however, there is currently no commercially available option for this diagnostic application (24).

In addition to *Borrelia* spp. and POWV, *I. scapularis* can transmit several other microorganisms, including *Anaplasma phagocytophilum*, *Ehrlichia muris eauclairensis*, and *Babesia microti*. Infection of ticks and humans with multiple pathogens is common (15, 25), and individuals with high tick exposure are frequently serologically positive for multiple organisms (26). *A. phagocytophilum* is a Gram-negative bacterium that infects granulocytes and can be diagnosed by serology, PCR, or identification of morulae on blood smears (27, 28). Symptoms of anaplasmosis, including fevers, headache, and malaise, are typically mild, and a rash can occur, which may indicate coinfection with *Borrelia* spp. *E. muris eauclairensis* is a recently recognized Gram-negative bacterium with an uncertain cellular target that generally causes mild disease (ehrlichiosis) (29, 30). *B. microti* is an apicomplexan protozoan that infects red blood cells and can be diagnosed by blood smears, serology, or PCR (31, 32). Babesiosis can cause severe disease due to hemolytic anemia and complications due to splenomegaly. Other pathogens with less certain clinical significance have also been associated with *I. scapularis* (33).

The primary objective of this study is to evaluate the utility of detecting *B. burgdorferi* and POWV in histologic sections of tick bite-associated skin biopsies. Precise localization of pathogens in tissue sections provides complementary information to molecular testing of homogenized samples, particularly in the assessment of potential molecular false positives. In addition, spatial resolution is critical for investigating pathogenesis in human tissues. To accomplish this goal, we have assembled a cohort of cases from a large academic medical center from a Lyme- and POWV-endemic region in the Northeastern USA and utilized a combination of stains, including hematoxylin and eosin (H&E), Warthin–Starry, spirochete immunohistochemistry (IHC), and POWV ISH. This comprehensive approach aims to provide valuable insights into enhancing diagnostic strategies for tick-borne diseases.

## RESULTS

A total of 36 biopsy cases were included in this study, encompassing tick bite-associated skin biopsies collected from 2010 to 2023 (Tables 1 and 2). The median age of the patients was 68 years, and the cohort was nearly equally split between males (58%) and females (42%). Three patients presented with skin lesions indicative of ECM, a characteristic symptom of Lyme and other tick-borne diseases, and seven patients were previously diagnosed with Lyme disease 1 or more years prior to their current presentation. Serologic screening for Lyme disease was positive in 6/18 (33%) cases submitted for testing, including two patients with ECM. One patient presented with subacute encephalopathy consistent with Lyme meningitis (CSF IgG positive). Another patient presented with altered mental status and was diagnosed with Powassan encephalitis by positive CSF POWV IgM and plaque reduction neutralization testing (PRNT) performed at the CDC. Additional tick-borne pathogen testing was performed for four patients, one of which was positive for *Anaplasma* IgM. Doxycycline was prescribed to 29/33 (88%) patients with available clinical data, 11 (38%) of which received 10–28 day courses, and the remaining 18 (62%) were treated with a single 200 mg prophylactic dose. In addition, one patient with doxycycline allergy was treated with a 14-day course of ceftriaxone and amoxicillin–clavulanic acid, and the POWV-positive patient was treated with vancomycin, ceftriaxone, ampicillin, and acyclovir for suspected infectious meningoencephalitis.

**TABLE 1:** Summary of patient demographics, clinical history, laboratory testing, and treatment^*d*^

Clinical characteristics		Skin biopsies (*n* = 36)	Whole ticks (*n* = 14)^*a*^
Demographics	Median [IQR] age in years	68 [58–75]	67 [59–73]
	Female sex, n (%)	15/36 (42%)	7/12 (58%)
Clinical history	ECM lesion, n (%)	3/36 (8%)	2/12 (17%)
	Prior history of Lyme, n (%)	7/36 (19%)	1/12 (8%)
Laboratory testing^*b*^	Positive Lyme serology/western blot, n (%)	6/18 (33%)	0/4 (0%)
	Positive Powassan PCR/ serology, n (%)	1/1 (100%)	−
	Positive *Anaplasma* PCR/serology, n (%)	1/4 (25%)	1/2 (50%)
	Positive *Babesia* PCR/ serology/blood smear, n (%)	0/4 (0%)	0/2 (0%)
Treatment^*c*^	Doxycycline prescribed, n (%)	29/33 (88%)	9/11 (82%)

^*a*^ Clinical data were not available for two out of 14 patients with whole ticks, and percentage values for each characteristic reflect the proportion of cases out of 12 with available clinical data.

^*b*^ Values for Lyme, Powassan, *Anaplasma*, and *Babesia* testing reflect the percentage of positive results divided by the total number of specimens submitted for pathogen-specific testing.

^*c*^ Information regarding treatment was only available for 33 patients with skin biopsies and 11 with whole tick specimens. Two additional patients were treated with non-doxycycline antibiotics due either to allergy or for treatment of suspected viral meningoencephalitis.

^*d*^ ECM, erythema chronicum migrans; IQR, interquartile range; "––" indicates testing was not attempted.

**TABLE 2:** Clinical and laboratory features and histological staining results for individual cases^*a*^^,*b*,*c*,*d*^

Specimen type	Case #	Clinical and laboratory characteristics					Histological staining results		
		ECM	Lyme testing	Other testing	Doxycycline	*Ixodes* tick	WS stain	Spirochete IHC	Powv ISH
Tick-bite skin biopsies	1	−			+	+	−	−	−
	2	−			+	n/a	−	−	−
	3	−	−		+	n/a	−	−	−
	4	−			+	+	−	−	−
	5	−			−	n/a	−	−	−
	6	+	−		+	n/a	−	−	−
	7	−			+	+	−	−	−
	8	−			n/a	n/a	−	−	−
	9	−	−		+	n/a	−	−	−
	10	−			−	n/a	−	−	−
	11	−			+	n/a	−	−	−
	12	−			+	+	−	−	−
	13	−			+	n/a	−	−	−
	14	−			+	n/a	−	−	−
	15	−	−	*Anaplasma* IgM+, PCR−, *Babesia* PCR/Smear−	+	n/a	−	−	−
	16	−			+	+	−	−	−
	17	−			+	n/a	−	−	−
	18	−			+	+	−	−	−
	19	−	−		+	+	−	−	−
	20	−	−		+	n/a	−	−	−
	21	−			+	+	−	−	−
	22	−	−		+	+	−	−	−
	23	−	−		+	+	−	−	−
	24	−	−		+	+	−	−	−
	25	−	−		+	+	−	−	−
	26	−			n/a	n/a	−	−	−
	27	−	−		n/a	n/a	−	−	−
	28	−			+	n/a	−	−	−
	29	−			+	+	−	−	−
	30	−	−	POWV IgM/PRNT+	−	n/a	−	−	−
	31	−	+	*Anaplasma* PCR−, *Babesia* smear−	+	n/a	−	−	−
	32	−	+		+	n/a	−	−	−
	33	+	+		+	n/a	−	−	−
	34	−	+	*Anaplasma* PCR−, *Babesia* PCR/Serology−	+	n/a	−	−	−
	35	+	+	*Anaplasma* PCR−, *Babesia* PCR−	+	n/a	−	−	−
	36	−	+		−	n/a	−	−	−
Whole ticks	1	n/a	n/a	n/a	n/a	+	−	−	−
	2	n/a	n/a	n/a	n/a	+	−	−	−
	3	−			+	+	−	−	−
	4	−	−	*Anaplasma* PCR+, *Babesia* PCR/Serology/Smear−	+	+	−	−	−
	5	−			n/a	−	−	−	−
	6	−			+	−	−	−	−
	7	−			+	+	+	+	−
	8	−	−		+	+	−	−	−
	9	+			+	−	−	−	−
	10	−			−	−	−	−	−
	11	+			+	+	+	+	−
	12	−	−		+	+	−	−	−
	13	−	−	*Anaplasma* PCR−, *Babesia* PCR−	+	+	−	−	−
	14	−			−	+	−	−	−

^*a*^ Empty cells indicate testing not performed.

^*b*^ n/a indicates data are unavailable.

^*c*^ “+” = positive/present, “−“ = negative/absent.

^*d*^ ECM, erythema chronicum migrans; WS, Warthin––Starry; IHC, immunohistochemistry; ISH, in-situ hybridization; PRNT, plaque reduction neutralization test.

**Fig 1:**
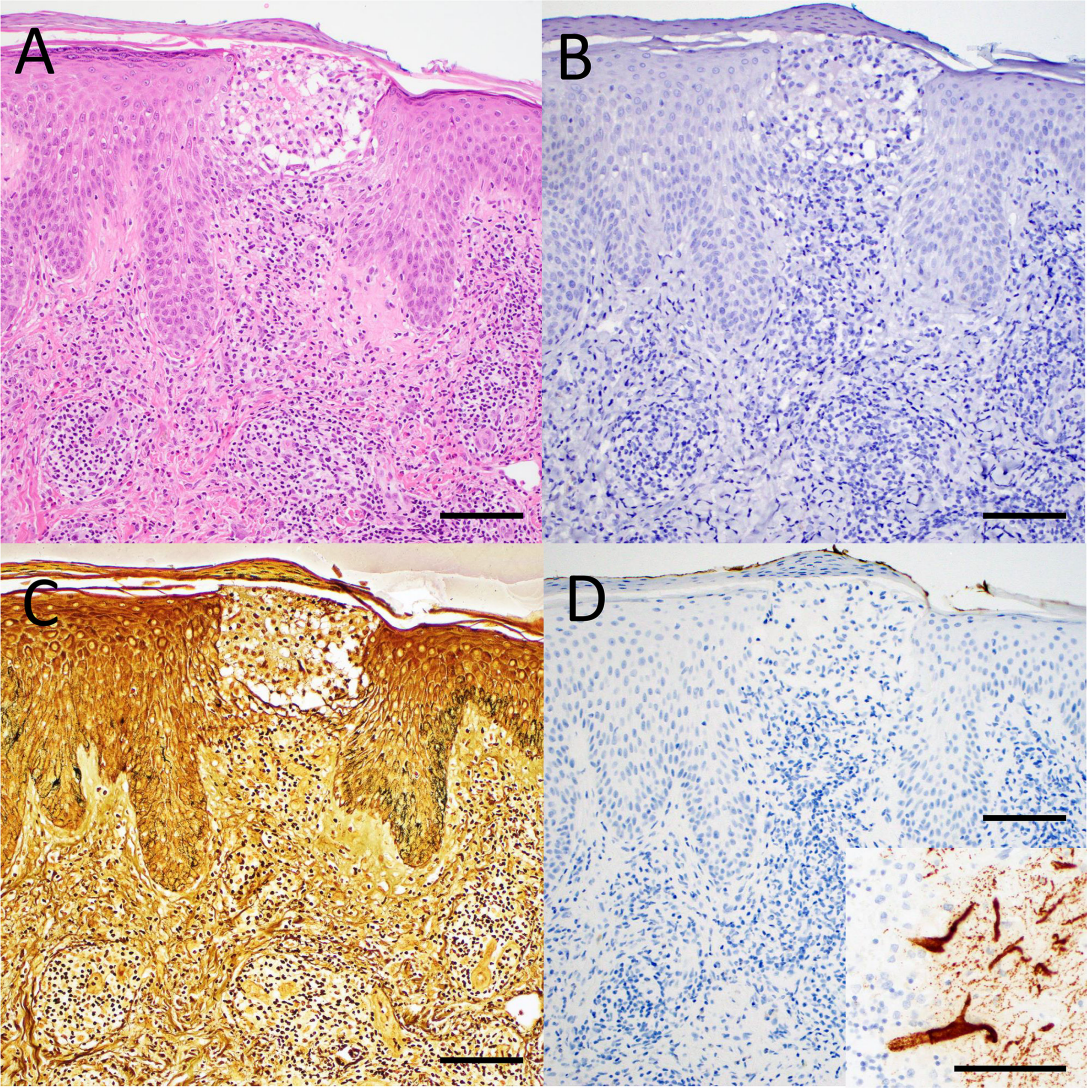
Evaluation of tick-associated skin biopsies from human patients. (**A**) The hematoxylin and eosin-stained section shows the site of a tick bite with excoriation and the characteristic perivascular lymphohistiocytic infiltrate with scattered eosinophils. (**B**) Spirochete immunohistochemistry was negative for bacteria in all cases. (**C**) Warthin–Starry staining was negative for bacteria in all cases. (**D**) Powassan virus *in situ* hybridization was negative in all cases. Staining (brown) in Purkinje neurons was observed in a positive control brain section from a fatal case of Powassan encephalitis (inset). Scale bars are 50 µm in all panels.

H&E-stained sections revealed ulceration and chitinous material consistent with retained tick mouth parts in 20/36 (56%) cases (Table 3). Typical features associated with arthropod assault reaction were present, including a lymphohistiocytic perivascular interstitial infiltrate in all cases (Fig. 1A), abundant eosinophilic infiltrate in 19 (53%) cases, prominent fibrin thrombi in 4 (11%) cases, abundant necrosis in 2 (6%) cases, and abscess in 1 (3%) case. No bacteria were identified by spirochete IHC (Fig. 1B) or Warthin–Starry stains (Fig. 1C), and POWV ISH was negative in all cases (Fig. 1D).

**TABLE 3:** Summary of tick identification and histological features^*b*^

Pathology		Skin biopsies (*n* = 36)	Whole ticks (*n* = 14)
Tick species	*Ixodes scapularis* group, n (%)	13 (36%)	10 (71%)
	*Dermacentor variabilis*, n (%)	0 (0%)	4 (29%)
	Unknown, n (%)^*a*^	23 (64%)	0 (0%)
	Tick parts seen on histology, n (%)	20 (56%)	14 (100%)
	Perivascular interstitial infiltrate, n (%)	36 (100%)	-
Histology	Eosinophilic infiltrate, n (%)	19 (53%)	-
	Fibrin thrombi, n (%)	4 (11%)	-
	Necrosis, n (%)	2 (6%)	-
	Abscess, n (%)	1 (3%)	-
Pathogen stains	Positive WS stain, n (%)	0 (0%)	2 (14%)
	Positive spirochete IHC, n (%)	0 (0%)	2 (14%)
	Positive Powassan ISH, n (%)	0 (0%)	0 (0%)

^*a*^ For a subset of tick-associated skin biopsies, ticks were not submitted for gross evaluation precluding species identification.

^*b*^ WS, Warthin–Starry; IHC, immunohistochemistry; ISH, *in-situ* hybridization; “−,” histological features not applicable.

**Fig 2:**
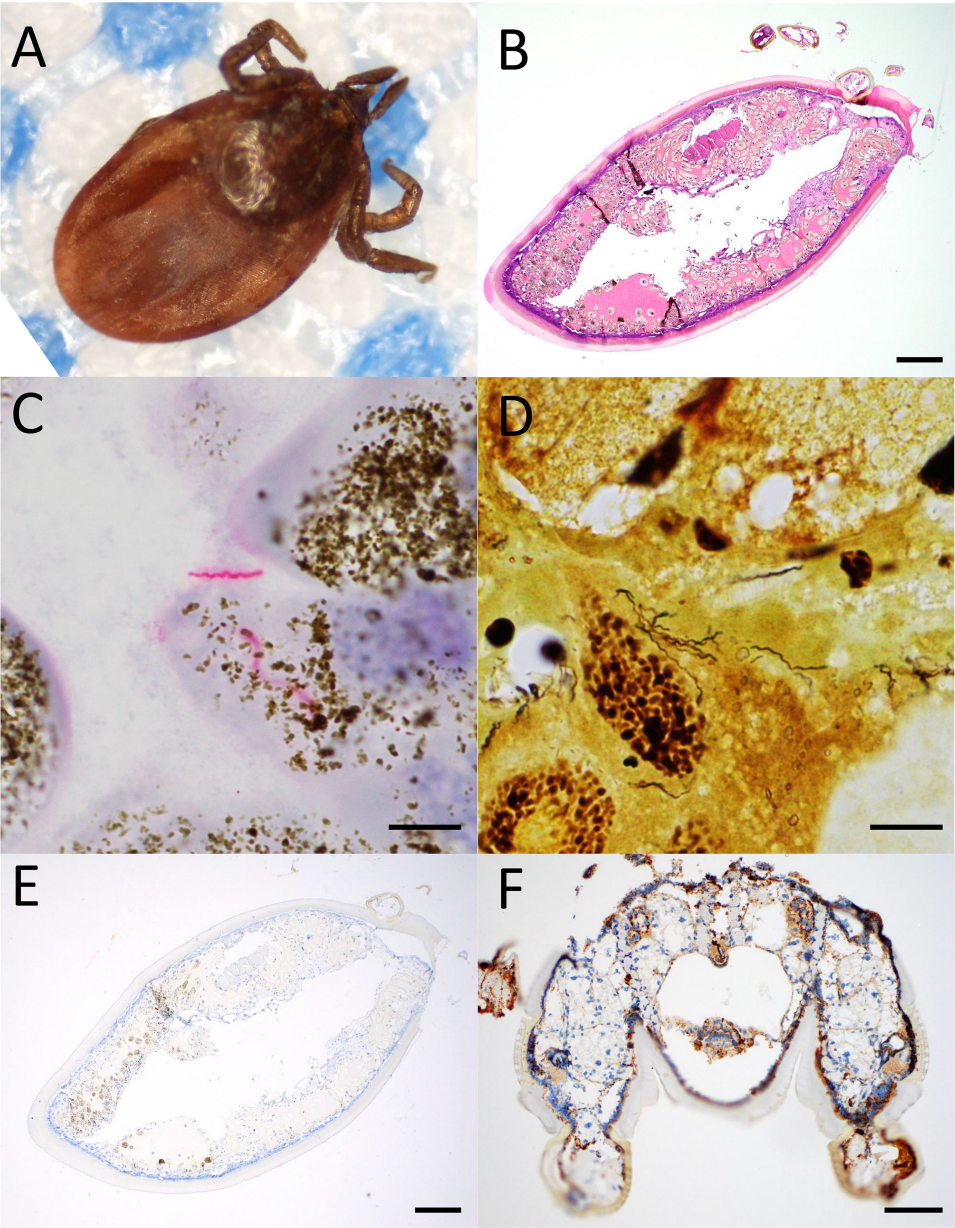
Evaluation of whole tick specimens collected from human patients. (**A**) An intact partially engorged adult female *Ixodes scapularis* is readily identified by red/orange body, black scutum, and U-shaped anal groove (not shown). (**B**) Sagittal section of *I. scapularis* stained with hematoxylin and eosin shows outer cuticle, underlying skeletal muscle, and inner gut. (**C**) Spirochete immunohistochemistry highlights scattered corkscrew-shaped bacteria (red) in gut adjacent to hemosiderin (brown). (**D**) Warthin−Starry highlights numerous long, corkscrew-shaped bacteria (black) in the gut. (**E**) Powassan virus RNA *in situ* hybridization is negative in all patient-derived ticks. (**F**) Powassan virus RNA *in situ* hybridization shows staining (brown) in a positive control female nymph tick experimentally infected with NFS001 strain and originally isolated from ticks collected on Nantucket Island. Virus is apparent in the hypodermis, epithelial cells of the gut caeca, and in some hemocytes. Scale bars are 200 µm for panels B and E, 100 µm for panel F, and 5 µm for panels C and D.

To ensure that our pathogen-targeted stains were optimized for FFPE specimens, we also tested tissue sections from whole ticks that had been removed from patients and submitted for gross identification. Among 14 whole tick specimens collected from 2019 to 2023, 10 (71%) were grossly identified as *Ixodes* spp. (i.e., *I. scapularis* group) (Fig. 2A) and four (29%) as *Dermacentor* spp. (i.e., *D. variabilis*; dog tick) (Tables 2 and 3). All *Ixodes* spp. ticks were adult females, and the majority (70%) exhibited at least partial engorgement indicative of >24 h attachment (Table 4). *Dermacentor* spp. ticks included two adult females and one adult male with minimal engorgement and one female nymph that was partially engorged. Following formalin fixation and standard tissue processing, whole ticks were sagittally oriented and embedded in paraffin, yielding sections showing cuticle, skeletal muscle, gut, and salivary glands (Fig. 2B). Spirochete IHC utilizing an anti-*Treponema pallidum* antibody with cross-reactivity to *Borrelia* spp. was positive in two out of 14 (14%) whole tick specimens (both adult female *I. scapularis* groups), highlighting numerous corkscrew-shaped bacteria throughout the tick gut adjacent to hemosiderin, consistent with recent feeding, as well as in salivary glands (Fig. 2C). Warthin–Starry silver staining produced results concordant with spirochete IHC, with numerous organisms identified in the same two cases (Fig. 2D). While one of the spirochete-positive ticks was from a patient with suspected ECM, the other spirochete-positive tick was from a patient who did not have ECM and a different patient with suspected ECM brought in a *Dermacentor* spp. tick that did not stain positive for spirochetes. Thus, as expected, there was low concordance between the results of tick testing and clinical disease. The presence of POWV RNA was assessed by *in situ* hybridization staining using custom probes broadly targeting the genome of a 2018 POWV lineage II strain from Massachusetts (34). POWV ISH was negative in all tick samples removed from patients (Fig. 2E). Experimentally infected positive control ticks exhibited viral RNA in the hypodermis, epithelial cells of the gut caeca, and in some hemocytes (Fig. 2F).

**TABLE 4:** Comparison of *Ixodes* and non-*Ixodes* (*Dermacentor*) whole tick specimens^*d*^

		*Ixodes* ticks (*n* = 10)	Non-*Ixodes* (*Dermacentor*) ticks (*n* = 4)
Clinical history^*a*^	Erythema chronicum migrans, n (%)	1/8 (13%)	1/4 (25%)
	Positive Lyme testing, n (%)	0/4 (0%)	-^*c*^
	Doxycycline, n (%)	7/8 (88%)	2/3 (67%)
Tick gross features	Adult female, n (%)	10/10 (100%)	2/4 (50%)
	Engorged, n (%)^*b*^	7/10 (70%)	1/4 (25%)
Histological stains	Positive WS stain, n (%)	2/10 (20%)	0/4 (0%)
	Positive spirochete IHC, n (%)	2/10 (20%)	0/4 (0%)
	Positive Powassan ISH, n (%)	0/10 (0%)	0/4 (0%)

^*a*^ Clinical data were not available for 2/10 *Ixodes* ticks.

^*b*^ Ticks with estimated >1 day (minimal) engorgement.

^*c*^ Indicates no laboratory testing was attempted.

^*d*^ WS, Warthin–Starry; IHC, immunohistochemistry; ISH, in-situ hybridization.

## DISCUSSION

Diagnosis of tick-borne infections is complex, requiring location-specific strategies due to geographic variation of tick species and associated pathogens. In this study, from the Northeastern USA, we assembled a cohort of 36 patients who presented with tick bites or rashes and underwent skin biopsy. We did not detect spirochetes by IHC in any of the specimens, despite the fact that the majority of specimens (56%) had retained tick mouth parts, and six patients had positive Lyme testing. These results are consistent with prior studies, which have shown low to moderate yield for detection of *Borrelia* spp. from skin biopsies by PCR, culture, or direct visualization, particularly in asymptomatic patients without ECM (35, 36).

POWV ISH was negative in all 36 skin biopsy specimens, including one laboratory confirmed case, which is not surprising given our small sample size and unknown rate of human exposure to this pathogen. Exposure is likely much more common than currently appreciated, as cases of neuroinvasive disease are likely underrecognized due to limited availability of diagnostic testing, and individuals with non-neuroinvasive infections are even less likely to be identified due to the self-limited and non-specific nature of the symptoms. Understanding the incidence of exposure, full clinical spectrum of POWV, and pathophysiology of early infection is important, as mouse models have shown that POWV may be transmitted within as little as 15 min of tick attachment (17), and POWV RNA can be detected at the tick bite site (24).

In addition to skin biopsies, we also performed staining for spirochetes and POWV in 14 ticks removed from patients, which was undertaken for scientific rather than diagnostic applications. While patients who experience tick bites frequently request testing the tick itself for pathogens, the CDC recommends against this practice due to: (1) the potential to detect organisms that were not transmitted, leading to unnecessary treatment; (2) the possibility that the patient was infected from a different tick than the one sent for testing, leading to potential undertreatment; and (3) delay in treatment for symptomatic individuals while the testing results are pending (37). Clinical laboratories can, however, perform gross morphological evaluation to classify tick species and estimate the duration of host attachment (i.e., level of engorgement) (38), which can inform empiric treatment and prophylaxis. Two of 14 ticks (14.3%) were found to have histological evidence of spirochetes, which in context were most likely Lyme-disease causing *B. burgdorferi*. In both cases, spirochetes were detected in the gut and salivary glands of adult female *I. scapularis* group ticks (2/10, 20%). Warthin–Starry and spirochete IHC (with polyclonal anti-*T*. *pallidum* antibody) highlighted similar numbers of organisms in both specimens. In contrast, Galan et al. reported greater sensitivity of Warthin-Starry staining compared to IHC with a polyclonal anti-*B*. *burgdorferi* antibody, although direct immunofluorescence staining with the same antibody used for IHC had even higher sensitivity than Warthin-Starry staining (39). In our study, spirochete IHC was easier to interpret, due to the red, slightly thicker appearance of organisms against a blue background. Warthin-Starry staining shows thinner dark-brown/black organisms against a yellow background, frequently with substantial background signal. While neither stain is specific for *Borrelia* spp., the association with tick exposure can help support a diagnosis of Lyme disease over infections with other spirochetes including *T. pallidum* and *Leptospira* spp.

POWV ISH was negative in all 14 ticks removed from patients, 10 of which were *Ixodes* spp. Our POWV ISH assay was confirmed to detect viral RNA both in experimentally infected ticks and patient-derived brain tissue, indicating that samples from this study were truly negative or at least below the level of detection of this assay. These results are not surprising given the small sample size since POWV is generally found in <5% of *I. scapularis* ticks (13–15).

The majority of patients in this study received doxycycline for treatment of presumed Lyme disease or for prophylaxis. This is in alignment with CDC recommendations for a single prophylactic dose in highly endemic areas aiming to reduce the risk of acquiring Lyme disease following a high-risk tick bite (40). However, concerns for antibiotic overuse have been raised, with >80% of patients receiving unnecessary antibiotics for a misdiagnosis of Lyme disease in some settings (41). Two ticks removed from patients in our study were confirmed to have spirochetes by IHC and Warthin–Starry staining. One spirochete-positive tick was associated with suspected ECM, suggestive of early localized Lyme disease, although the erythema noted clinically may be directly attributable to tick attachment. This adult female tick was noted to be minimally engorged, consistent with a shorter attachment time and a low likelihood of *Borrelia* spp. transmission from this particular tick. Instead, the patient’s symptoms may have been due to infection from other recent tick bites. The patient was appropriately treated with a full course of doxycycline. The other patient presented with an engorged adult female tick and no symptoms, which, per guidelines, would warrant a prophylactic dose of doxycycline, irrespective of the tick testing results. Finally, we note that a patient with suspected ECM brought in a *Dermacentor* spp. tick that did not stain positive for spirochetes, and the patient received a full course of doxycycline based on symptoms of early Lyme disease, irrespective of the tick identification results. Thus, overall, our results support the lack of utility in tick testing to guide clinical management.

While this study demonstrates the feasibility of histological detection of tick-borne pathogens, there are several limitations to be considered. First, the low number of laboratory-confirmed Lyme disease and POWV encephalitis cases introduces a significant constraint, hindering a comprehensive assessment of the clinical utility of IHC and ISH testing, and precludes the calculation of sensitivity and specificity. However, several patients in this cohort had a prior history of laboratory confirmed Lyme disease, and one patient was subsequently diagnosed with POWV encephalitis several years after negative skin biopsy testing, indicating the high risk for tick-borne infections in this cohort. Sample size is the biggest limitation for POWV, which is only expected to be present in 1–4% of ticks, and it is unknown whether it would be found in skin biopsies (13–15). The presence of spirochetes in ticks, while suggestive, cannot definitively confirm *B. burgdorferi* infection, particularly for minimally engorged ticks which may not have been attached a sufficient period to facilitate transmission. Moreover, the potential for cross-reactivity of antibodies with related bacteria raises questions about the specificity of the spirochete IHC (42). It is important to note, however, that serology shares this cross-reactivity limitation, compromising the test specificity, particularly in non-endemic areas and in patients with low pretest probability. Additionally, the background rate of seropositivity in endemic regions can reach up to 4%, further impacting the reliability of a positive serology result (43). POWV RNA ISH is unlikely to have been falsely negative due to a mismatch between probes and the target sequence since the assay uses 20 sets of probes targeting a clinically derived genome from the same geographic region from 2018, with confirmed cross-reactivity to the experimentally infected ticks using a strain isolated in 1996. Lastly, ticks in this study consisted predominantly of adult female ticks, while the smaller and less commonly identified female nymphs are more likely to transmit infections and cause disease. These acknowledged limitations reinforce the complexity of tick-borne diseases and highlight the need for further research with larger laboratory-confirmed cohorts to validate the findings and explore alternative diagnostic strategies.

While we cannot make definitive conclusions regarding the utility of Warthin–Starry and spirochete IHC for detecting spirochetes or POWV ISH for detected POWV in tick bite skin biopsies, the lack of positive staining in our highly enriched real-world cohort does not provide strong evidence in favor of routine clinical use. Our study does demonstrate the feasibility of utilizing histologic methods for spirochete detection in whole ticks and supports the notion that testing should not be done for clinical purposes. In concordance with the published literature, we found a poor correlation between detection of Lyme-associated spirochetes and ECM (44, 45). While the study primarily focuses on *B. burgdorferi*, the presence of *Anaplasma* positive patients raises questions about the potential application of *in situ* hybridization for diagnosis of anaplasmosis. Neither H&E nor Warthin–Starry stains are optimal for identification of *Anaplasma* spp., which are most commonly seen in cytoplasm of granulocytes on blood smears, but can also be highlighted by targeted IHC (46). Routine histologic staining of *Dermacentor* spp. ticks is not recommended for *Borrelia* spp. or POWV, as these are not known to be associated pathogens, although more relevant targets, such as *Francisella tularensis* and *Rickettsia* spp., could be considered. These findings underscore the challenges of diagnosing tick-borne diseases and emphasize the importance of tailored diagnostic approaches to enhance accuracy and clinical relevance.

## MATERIALS AND METHODS

This study was approved by the Mass General Brigham Institutional Review Board under an excess tissue, waived consent protocol. Cases were retrospectively identified for inclusion by searching the Brigham and Women’s Hospital (BWH) pathology reports from 2010 to 2023. Electronic medical records were reviewed for relevant clinical history, laboratory test results, and treatments administered to the patients in connection with tick-borne diseases. Tick-associated skin biopsy cases were identified through keyword searches,including “punctate,” “punctum,” “tick,” “mouthparts,” “mouth,” and “*Ixodes*.” In addition, skin biopsies were identified from patients with laboratory-confirmed diagnoses of Lyme disease (serum IgG and/or IgM positive) or Powassan disease (serum/CSF IgM, plaque reduction neutralization test, RT-PCR, or metagenomic next-generation sequencing positive). All cases were included with sufficient formalin-fixed paraffin-embedded (FFPE) tissue available to prepare ten 5-micrometer sections. Intact ticks received for gross identification by the BWH dermatopathology service or the BWH clinical microbiology laboratory from 2019 to 2023 were subsequently fixed in 10% formalin for >24 h, followed by standard tissue processing and paraffin embedding. Ticks were oriented sagittally or coronally and cut in 5 µm sections. Skin biopsy and tick sections were stained with H&E, Warthin–Starry silver staining, spirochete immunohistochemistry (IHC), and POWV RNA ISH. Warthin–Starry staining was performed using the Artisan Link Pro Automatic Staining Platform (Agilent Technologies, Santa Clara, CA) with the Artisan Warthin–Starry Stain Kit (AR181; Agilent Technologies). Spirochete IHC used a rabbit polyclonal anti-*Treponema pallidum* antibody (CP135C; Biocare Medical, Pacheco, CA) at 1:200 dilution, following antigen retrieval using citrate buffer (pH 6.0) and pressure cooker. PowerVision poly-alkaline phosphatase anti-rabbit IgG (PV6133; Leica Biosystems, Nussloch, Germany) was used for detection, which produced a red signal. POWV RNA ISH was performed on the Leica BOND-III Automated Staining System (Leica Biosystems) using custom-designed RNAScope probes V-POWV-pp-C1 (1123998-C1, Advanced Cell Diagnostics, Newark, CA) targeting 748–7571 base pairs of GenBank accession MT996002.1 according to manufacturer protocols (34, 47, 48). In brief, FFPE tissue sections were baked for 30 min at 60°C and deparaffinized (AR9222; Leica Biosystems) prior to staining. Target retrieval was performed with Bond ER Solution 2 (AR9640; Leica Biosystems) for 15 min at 95°C, followed by room-temperature RNAScope enzyme III treatment with BOND RNAScope protease (AR9733; Leica Biosystems) for 15 min, followed by RNAscope H_2_O_2_ (DS9790; Leica Biosystems) for 10 min. Slides were then incubated with probes for 120 min at room temperature, followed by RNAscope DAB ISH Protocol 2 (DS9790; Leica Biosystems) to produce a brown signal and counterstained with hematoxylin and RNAscope bluing.
